# Slope walking causes short-term changes in soleus H-reflex excitability

**DOI:** 10.14814/phy2.12308

**Published:** 2015-03-05

**Authors:** Manning J Sabatier, Wesley Wedewer, Ben Barton, Eric Henderson, John T Murphy, Kar Ou

**Affiliations:** Department of Rehabilitation Medicine, Emory University School of MedicineAtlanta, Georgia

**Keywords:** Homosynaptic depression, locomotion, rate-dependent depression, spinal excitability, treadmill

## Abstract

The purpose of this study was to test the hypothesis that downslope treadmill walking decreases spinal excitability. Soleus H-reflexes were measured in sixteen adults on 3 days. Measurements were taken before and twice after 20 min of treadmill walking at 2.5 mph (starting at 10 and 45 min post). Participants walked on a different slope each day [level (Lv), upslope (Us) or downslope (Ds)]. The tibial nerve was electrically stimulated with a range of intensities to construct the M-response and H-reflex curves. Maximum evoked responses (*H*_max_ and *M*_max_) and slopes of the ascending limbs (Hslp and Mslp) of the curves were evaluated. Rate-dependent depression (RDD) was measured as the % depression of the H-reflex when measured at a rate of 1.0 Hz versus 0.1 Hz. Heart rate (HR), blood pressure (BP), and ratings of perceived exertion (RPE) were measured during walking. Ds and Lv walking reduced the *H*_max_/*M*_max_ ratio (*P* = 0.001 & *P* = 0.02), although the reduction was larger for Ds walking (29.3 ± 6.2% vs. 6.8 ± 5.2%, *P* = 0.02). The reduction associated with Ds walking was correlated with physical activity level as measured via questionnaire (*r* = −0.52, *P* = 0.04). Us walking caused an increase in the Hslp/Mslp ratio (*P* = 0.03) and a decrease in RDD (*P* = 0.04). These changes recovered by 45 min. Exercise HR and BP were highest during Us walking. RPE was greater during Ds and Us walking compared to Lv walking, but did not exceed “Fairly light” for Ds walking. In conclusion, in healthy adults treadmill walking has a short-term effect on soleus H-reflex excitability that is determined by the slope of the treadmill surface.

## Introduction

The spinal cord is a major locus of activity-dependent neural plasticity associated with motor learning and skilled performance improvements (Windhorst 1996; Wolpaw and Tennissen 2001). Activity-dependent spinal plasticity can be evoked in the short-term and the long-term, and spinal excitability reflects the regular patterns of motor activity in which people engage (Zehr 2002). For example, soleus H-reflexes are smaller in explosively trained athletes (Casabona et al. 1990) and skilled ballet dancers (Nielsen et al. 1993), and larger in endurance trained athletes, compared to other athletes and to untrained controls (Casabona et al. 1990; Maffiuletti et al. 2001). Walking is a ubiquitous and fundamental rhythmic activity that supports independence and quality of life (Yildiz 2012) and also has been found to evoke spinal plasticity. For example, a recent study reported that 30 min of level treadmill walking causes short-term H-reflex depression in healthy adults (Thompson et al. 2006). Another study found that 20 min of over-ground walking caused an increase in rate-dependent depression of H-reflexes, a form of presynaptic inhibition (Phadke et al. 2009). However, it is not known if the response of the H-reflex pathway could be augmented by changing the parameters of the walking task, for example, surface slope.

During downslope (Ds), upslope (Us), and level (Lv) walking lower extremity muscles express unique patterns of electromyographic (EMG) activity that reflect differences in motor output and afferent feedback with different slopes (Akima et al. 2005; Gregor et al. 2006; Lay et al. 2007). For example, an increase in extensor muscle EMG activity during Us walking, and a decrease during Ds walking, has been reported for both quadrupeds (Gregor et al. 2006; Sabatier et al. 2011) and humans (Lay et al. 2007; Franz and Kram 2012). Moreover, during Ds walking there is increased muscle length-dependent afferent feedback compared to Lv or Us walking due to increased reliance on eccentric muscle contractions (Kuster et al. 1995; Abelew et al. 2000; Gregor et al. 2001; McIntosh et al. 2006). Downslope walking might also require more cortical activity than either Lv or Us walking due to this reliance on eccentric muscle contractions (Fang et al. 2001, 2004). This might cause an increase in cortical mediated spinal inhibition that would manifest as a reduction in spinal excitability (Wolpaw 2007). Another way Ds walking could affect reduced spinal excitability is through activation of the Ia spinal reflex arc. For example, activation of the Ia spinal reflex arc through stimulation of muscle spindles by vibration results in decreased spinal excitability (Shinohara 2005). Thus, Ds walking, through a more natural form of persistent activation of the spinal Ia reflex arc, may have a similar effect. Finally, Ds walking is less metabolically demanding and evokes a smaller cardiovascular response than level or Us walking (Knuttgen et al. 1971; Navalta et al. 2004). Therefore, as a potential adjunct for exercise prescription, Ds walking could be more accommodating for people with reduced exercise capacity.

It is also possible that Us walking might have a unique ability to evoke spinal plasticity. During Us walking the body's center of mass is moved vertically, against gravity, and there is increased motor unit recruitment in lower extremity muscles compared to Lv and Ds walking (Lay et al. 2007; Franz and Kram 2012). Increased effort required for Us walking would be expected to promote increased serotonergic and noradrenergic signaling (Aston-Jones et al. 2000; Jacobs et al. 2002), potentially leading to a change in spinal excitability. An increase in spinal excitability would facilitate muscle stiffness and force transmission in the lower extremity extensor muscles to serve the Us walking movement pattern. At this time, whether or not walking slope modulates the effect of walking on spinal synaptic transmission remains unknown.

The purpose of this study was to test the hypothesis that Ds walking evokes a decrease in soleus H-reflex excitability. This study also hypothesized an increase in soleus H-reflex excitability as a result of Us walking. Soleus H-reflexes were measured, whereas participants were resting in the recumbent seated position both before and after treadmill slope walking.

## Methods

### Participants

Sixteen participants (nine men and seven women) between the ages of 23 and 44, who did not participate in competitive sports, and with no neurologic disease or injury were tested on 3 days within a period of 1 week. Participant characteristics were as follows (mean ± SD): age: 27.3 ± 6.0 years; height: 177.8 ± 10.5 cm; mass: 76.3 ± 15.6 kg; BMI: 23.9 ± 3.4 kg/m^2^. All participants were tested before and after treadmill walking. However, four of these participants were tested for only the pre and the first postwalking time point. Each visit started within the same 1-h window of time to avoid the effects of diurnal variation in reflex size (Wolpaw and Seegal 1982; Lagerquist et al. 2006; Thompson et al. 2013). All participants were familiar with treadmill walking. The right leg was tested in all but one participant, who asked for the left leg to be tested. Written informed consent was obtained from all participants, and the study was approved by the Institutional Review Board of Emory University.

### Procedures

Physical activity (PA) during the week prior to testing was assessed with the 7-day PA recall questionnaire (Blair et al. 1985; Motl et al. 2003b; Cureton et al. 2009). There was a range of values for PA (40–425 kJ/day), but the group average (212 ± 113 kJ/day) was similar to healthy college-aged nonathletes as reported in other studies (Motl et al. 2003b; Cureton et al. 2009). Participants walked at a slow speed, 4.0 km/h (2.5 mph), on a Sole Fitness F85 Folding Treadmill (Niagra Falls, ON) downslope (−8.5°, −15%), level (0%), and upslope (2.9−8.5°, i.e., 5–15%), for 20 min. Duration was selected based on previous reports of altered H-reflex responses with other activities (treadmill running (Bulbulian and Darabos 1986), cycle ergometry (Motl et al. 2006)). For Us walking the treadmill slope changed every 5 min. The order of slopes was 2.9°, 8.5°, 5.7°, and 2.9°. This pattern was used to control for the much larger increase in effort that is associated with Us walking, and to ensure that all participants could maintain a full 20 min of uninterrupted Us walking (Navalta et al. 2004).

A different walking condition was tested for each test session. Upslope and Lv walking were randomized to days 1 and 2. Downslope walking was always reserved for day 3 because of the potential for delayed onset muscle soreness (Whitehead et al. 2001; Farr et al. 2002; Nottle and Nosaka 2005) that could have then affected walking and/or H-reflexes on the other 2 days of testing (Vangsgaard et al. 2013). None of our participants had ever engaged in uninterrupted Ds treadmill walking as used in this study. Therefore, to acclimate participants to the Ds walking task, each subject walked Ds for 3 min at the end of the day-2 session. We have previously found that this duration of Ds walking does not cause exercise-induced muscle injury or delayed onset muscle soreness (Sabatier and Black 2012). Prior to walking a wireless tri-axial accelerometer was strapped securely to the right ankle (BN-ACCL3; Biopac Systems Inc., Goleta, CA). Stride frequency was computed online for each step using the time period separating consecutive accelerations resulting from foot contact with the treadmill.

During the 5^th^, 10^th^, 15^th^, and 20^th^ min of walking heart rate was measured using a Polar Ft1 Heart Rate Monitor (Polar Electro Inc.; Lake Success, NY). Arterial pressure was measured at the brachial artery by manual sphygmomanometry using an appropriate-sized cuff (Perloff et al. 1993). Participants were also asked to rate their perceived exertion using the Borg scale, with 6 being “very, very light” to 19 being “very, very hard” (Borg 1978). After walking participants were immediately seated and prepared for post H-reflex testing. Heart rate and blood pressure were measured once more 5 min after walking stopped. H-reflex collection began again at 10 min (*n* = 16), and then again at 45 min (*n* = 12).

### Soleus H-reflexes

The H-reflex was measured at the soleus while the participant was seated in a semireclined position with the hip at 120° and the knee at 30°. The soleus was chosen because it is strongly modulated during slope walking (Lay et al. 2007). A band was secured around the legs at the distal thigh to prevent the legs from falling into external rotation or abduction. Electromyographic (EMG) activity was recorded from the soleus using bipolar electrodes (EL503; Biopac Systems Inc.) placed 2 cm apart along the posterior lateral aspect of the muscle, 2 cm inferior to the lower border of the lateral gastrocnemius (Basmajian and Blumenstein 1989). Identical day-to-day EMG and stimulation electrode placement was ensured by outlining electrode locations on the skin with a permanent marker. EMG signals were band-pass filtered (5–1000 Hz) and amplified by 2000 (BN-EMG2; Biopac Systems Inc.). Low impedance (<10 kΩ) was verified for all stimulating and recording electrodes using an electrode impedance meter (UFI MkIII Checktrode, Model 1089).

Reflexes were evoked by stimulating the tibial nerve in the popliteal fossa through a monopolar electrode (round, 2.5 cm) with the anode (square, 5 cm) placed above the patella. Both were self-adhering carbon rubber TENS/NMES electrodes (Medical Products Online, Danbury, CT). Cathode placement was determined prior to testing using a pen electrode (Model G.MPPE, Digitimer, Hertfordshire, AL7 3BE, England) to find the location yielding an H-reflex without an M-response and plantar flexion without eversion or inversion. Single, 1-msec rectangular pulses were delivered at pseudo-random intervals (5–8 sec in duration) using a constant-current electrical stimulator (STMISOLA; BIOPAC Systems Inc.) controlled with custom-written scripts in AcqKnowledge software (Biopac Systems Inc.).

The amplitude of the H-reflex depends on motoneuron pool excitability (as measured via background EMG activity) when the H-reflex is elicited. Therefore, when evaluating H-reflexes it is important to standardize for the level of motoneuron pool excitability (Schieppati 1987; Burke et al. 1989; Zehr 2002). In this study soleus background EMG activity was maintained at a constant low level as measured from participants during quiet standing (Thompson et al. 2009). The ankle was maintained at 10^o^ of plantar flexion using a foot brace that the subject contracted against to adjust soleus EMG activity to the required level. Prior to H-reflex testing participants stood quietly for 30 seconds while soleus and tibialis anterior EMG activity were measured. The average rectified soleus EMG activity was recorded. During collection of H-reflex recruitment curves participants were provided with visual feedback to maintain average rectified soleus EMG activity at standing level.

#### Spinal excitability

The H-reflex recruitment curve was acquired by progressively increasing the intensity of electrical stimulation in 0.5–1.0 mA increments to find the largest obtainable H-reflexes and M-responses, measured as the peak-to-peak amplitude of the raw EMG signal. The *H*_max_ was taken as the average of the largest three H-reflexes, and the *M*_max_ as the average of the largest three M-responses.

The M-response increases with increasing stimulus intensity, eventually reaching a plateau (referred to as *M*_max_ subsequently). *M*_max_ is an estimate of the motoneuron pool. Longitudinal studies generally express *H*_max_ as a percentage of the *M*_max_ to account for potential changes in ability to deliver current to the peripheral nerve across time and for differences in muscle geometry across subjects (Crone et al. 1999; Zehr 2002; Palmieri et al. 2004). This is also done to control for intersubject differences in the efficacy of nerve stimulation and total number of MUs accessible by nerve stimulation (Palmieri et al. 2004). Therefore, one expression of spinal excitability used in this study is the *H*_max_/*M*_max_ ratio.

A drawback to characterizing spinal excitability in the manner described above is that stimulating above motor threshold results in collision between orthodromic and antidromic impulse transmission. This may contribute to inter and intrasubject variability in *H*_max_, and to the decreased sensitivity of the H-reflex facilitation and inhibition at *H*_max_ (Crone et al. 1990). The rising slope of the H recruitment curve (Hslp) has been suggested as a good alternative because it is free of this collision effect (Funase et al. 1994). Also, in previous studies, an increase in spinal excitability was detected using the Hslp/Mslp approach, but not with the *H*_max_/*M*_max_ ratio (Kalmar and Cafarelli 1999; Walton et al. 2003). Therefore, in order to optimize this study's potential to detect an increase in spinal excitability the slope of the H-reflex recruitment curve was also evaluated. Recruitment curves were fitted with 8^th^ order polynomial transformations starting at the first response of the ascending limb using custom-written scripts in Microsoft Excel. R-squared values for raw data versus computed curves were high (mean ±SE, 0.96 ± 0.01). The ratio of the Hslp to the M recruitment curve slope (Mslp) was computed to standardize for motoneuron excitability. This renders the Hslp/Mslp ratio a metric of spinal excitability. Each slope was derived from the linear regression line that included values from the transformed curve between 25% and 75% of the *H*_max_ and *M*_max_, for the Hslp and Mslp, respectively.

#### Rate-dependent depression (RDD)

Rate-dependent depression (also known as postactivation depression) was measured as a segmental presynaptic mechanism of activity-dependent synaptic efficacy in the H-reflex pathway (Kohn et al. 1997; Hultborn and Nielsen 1998; Aymard et al. 2000). Ten H-reflexes were elicited at a low stimulus frequency of 0.1 Hz, and at the stimulus intensity that elicited an H-reflex that was between 20% of *M*_max_ and 50% of *H*_max_ (Sosnoff and Motl 2010). The H-reflex was elicited 11 times at the same stimulus intensity, but with a stimulus frequency of 1 Hz (i.e., high-frequency stimulation), and the last 10 H-reflexes of this series were averaged. The average amplitude of these H-reflexes is expressed as a percentage of the average H-reflex amplitude when evoked at a frequency of 0.1 Hz (Sosnoff and Motl 2010). Voluntary muscle contraction decreases RDD (Hultborn and Nielsen 1998). Therefore, participants were instructed to keep the leg at complete rest throughout RDD testing (Meunier et al. 2007; Lamy et al. 2009).

### Muscle soreness

Downslope walking is associated with more eccentric muscle contractile activity than Lv or Us walking (Abelew et al. 2000; Gregor et al. 2001; Akima et al. 2005). Because eccentric muscle contractions are associated with increased risk for delayed onset muscle soreness (DOMS) (Proske and Morgan 2001), Ds walking might cause DOMS. In fact, previous studies have used a variety of Ds locomotion patterns as a way to induce muscle soreness (Whitehead et al. 2001; Farr et al. 2002; Nottle and Nosaka 2005). Although no previous study to our knowledge has used the Ds walking pattern described in this study, we anticipated muscle soreness might result in lower extremity muscles after Ds walking. Therefore, muscle pain intensity in the lower extremities (anterior and posterior leg, anterior thigh) was assessed using a visual analogue scale (VAS). The VAS consists of a 10-cm line ranging from 0 (no pain) to 10 (worst pain imaginable). Participants rated pain intensity felt during daily life activity during the 4 days after the last session.

### Statistical analysis

This study used a repeated measures design with outcomes collected on all 3 days. The repeated measures design was modeled using a general linear model with slope as a fixed effect and random effects for subject nested within time point using R version 3.1.0 (The R Foundation for Statistical Computing) (Bates et al. 2014). If the interaction term for the model was significant, each post time point was compared to the pre time point using a model adjusted t-test where the standard errors and degrees of freedom were estimated from the model with restricted maximum likelihood methodology. Intraclass correlation coefficients were computed using Statistica data analysis software system (version 10, StatSoft, Inc., 2013) to evaluate day-to-day reliability of dependent measures. Linear correlation analysis was carried out to determine the relationship between variables of interest. The T-distribution was used to determine the statistical significance of correlations. The significance level was set at *P* ≤ 0.05 for all statistical tests.

## Results

**Figure 1:**
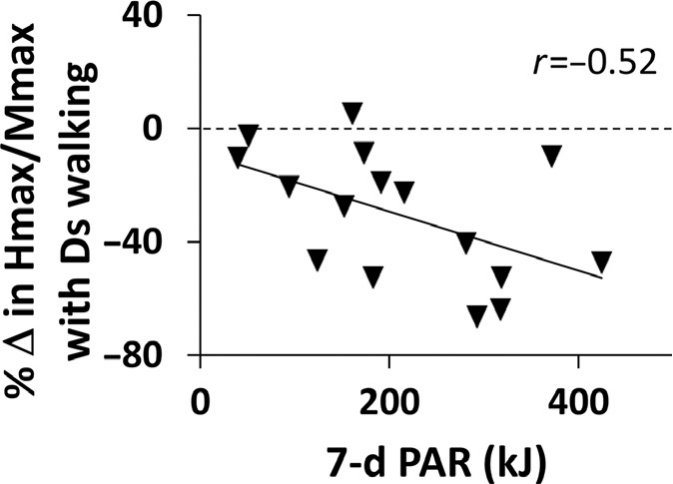
The depressive effect of Ds walking (ordinate) as a function of physical activity level (7-d PAR, abscissa) is illustrated in this scatterplot. The post-pre *H*_max_/*M*_max_ difference is expressed as a percentage of pre on the ordinate. A horizontal dashed line is aligned at zero on the ordinate, or an absence of change in *H*_max_/*M*_max_. Values below zero reflect a reduction in *H*_max_/*M*_max_ after Ds walking. There was a significant negative correlation between the percent change in *H*_max_/*M*_max_ with Ds walking and physical activity (*r* = −0.52, *P* = 0.04).

Study participants were healthy adults who did not participate in competitive sports. Nevertheless, we anticipated regular PA levels might vary significantly and contribute to variability in starting values of our dependent measures, or the way these values responded to slope walking. Therefore, this study also measured PA via questionnaire to determine if there are relationships between regular PA and our dependent measures. There was a significant negative correlation between the change in *H*_max_/*M*_max_ with Ds walking and PA (*r* = −0.52, *P* = 0.04, Fig.1). Therefore, participants who reported more PA had a larger reduction in *H*_max_/*M*_max_ after Ds walking. There was no correlation between PA and *H*_max_/*M*_max_ (*r* = 0.39, *P* = 0.20), Hslp/Mslp (*r* = 0.22, *P* = 0.49), or RDD (*r* = 0.06, *P* = 0.85). There was no correlation between PA and the change in *H*_max_/*M*_max_ for Lv (*r* = −0.14, *P* = 0.60) and Us walking (*r* = 0.11, *P* = 0.69). There were also no significant correlations between PA and the change in Hslp/Mslp (*r* = −0.26, *P* = 0.33; *r* = 0.01, *P* = 0.98; *r* = −0.07, *P* = 0.79) or RDD (*r* = 0.27, *P* = 0.31; *r* = −0.20, *P* = 0.45; *r* = 0.23, *P* = 0.39) with Ds, Lv or Us walking, respectively.

During treadmill walking there were several slope-related changes in measures associated with effort. Effort during slope walking was monitored by measuring blood pressure (BP), heart rate (HR) and ratings of perceived exertion (RPEs) (Table1). Heart rate was highest during Us walking, and not significantly different between Lv and Ds walking. An exception was after treadmill walking. Five minutes after cessation of both Ds and Us walking HR remained elevated compared to after Lv walking. Heart rate increased during the Ds walking bout and was highest during the 3^rd^ and 4^th^ epochs. Like HR, systolic blood pressure (SBP) was also highest during Us walking. However, SBP was similar between Lv and Ds walking. Diastolic blood pressure (DBP) was higher during Ds walking than during both Lv and Us walking. However, DBP fell to a lower level at 5 min after cessation of Ds walking compared to either Lv or Us walking. Ratings of perceived exertion were higher for both Ds and Us walking compared to Lv walking. For Ds walking RPE did not exceed 10 (9 was anchored as “Very light,” and 11 was “Fairly light”). Thus, although there was increased perceived effort associated with walking on either slope, it remaind light for Ds walking.

**Table 1:** Cardiovascular function and perceived exertion during each 5-min epoch of slope walking

	Pre	Treadmill walking				Post
		1–5 min	6–10 min	11–15 min	16–20 min	
HR						
Us	61 (10)	94 (14)	117 (23)	114 (21)	105 (17)	67 (13)
Lv	63 (11)	83 (13)*	87 (13)*	88 (14)*	87 (15)*	61 (10)
Ds	63 (9)	81 (12)*	84 (11)*	88 (10)*	87 (13)*	66 (11)*
SBP						
Us	117 (15)	123 (19)	133 (18)	125 (17)	119 (14)	119 (15)
Lv	113 (13)*	116 (16)*	112 (15)*	113 (16)*	114 (15)*	115 (13)
Ds	113 (10)	117 (13)*	117 (16)*	114 (15)*	116 (15)	114 (9)*
DBP						
Us	76 (9)	68 (11)	64 (13)	61 (10)	61 (11)	75 (11)
Lv	71 (9)*	66 (9)	65 (10)	64 (11)	63 (9)	74 (9)
Ds	71 (11)*	70 (9)#	68 (9)	69 (9)*#	69 (9)*#	71 (8)*#
RPE						
Us	–	7.8 (1.0)	12.0 (2.0)	10.7 (1.1)	9.3 (1.7)	–
Lv	–	7.3 (1.1)	7.6 (1.2)*	7.6 (1.2)*	7.6 (1.1)*	–
Ds	–	8.8 (1.6)*#	9.6 (2.0)*#	9.9 (2.0)*	10.0 (2.2)*	–

Values are reported as mean (SD).

SBP, systolic blood pressure; DBP, diastolic blood pressure.

* *P* < 0.05 vs. Us.

# *P* < 0.05 vs. Lv.

### Step cycle timing

Stride frequency was measured to determine if basic step cycle timing for walking at different slopes in this study is consistent with previous reports on the biomechanics of slope walking. Stride frequency was significantly greater for Ds walking (1.04 ± 0.07 Hz) compared to Lv (0.85 ± 0.02 Hz) and Us walking (0.83 ± 0.07 Hz), *P* ≤ 0.05 for all comparisons. These results are consistent with previous investigations of the biomechanics of walking on different slopes (Hunter et al. 2010; Franz and Kram 2012, 2013). Thus, although comprehensive biomechanical measures of lower body movements were not made here, the results of step cycle timing, effort and DOMS support the idea that this study compared well to the studies referenced above.

### Lower extremity muscle soreness

There were no reports of soreness or other discomfort after either Lv or Us walking. Although five participants reported no soreness, all other participants reported soreness ranging from 0.5 cm to 8.4 cm on the VAS scale after Ds walking. Soreness only occurred after Ds walking, and the majority of soreness was experienced in the tibialis anterior and the triceps surae (Table2). This effect subsided during the 4 days after Ds walking, supporting the notion that the Ds walking pattern used in this study involved more eccentric muscle contraction than either Lv or Us walking. Incidently, one subject also reported hip flexor soreness near the inguinal ligament. Another subject reported no soreness in the quadriceps, but rather a generalized feeling of fatigue in the legs.

**Table 2:** Muscle soreness after downslope walking

	Four days following Ds treadmill walking			
	24 h	48 h	72 h	96 h
Tibialis Anterior				
R	1.4 (2.3)	0.9 (1.6)	0.2 (0.4)*	0.0 (0.1)*
L	1.1 (1.8)	0.9 (1.9)	0.3 (0.6)*	0.1 (0.2)*
Triceps Surae				
R	1.4 (2.2)	0.9 (1.7)*	0.3 (0.7)*	0.1 (0.2)*
L	1.4 (2.3)	0.9 (1.9)*	0.3 (0.6)*	0.1 (0.2)*
Quadriceps				
R	0.3 (0.8)	0.2 (0.5)	0.1 (0.2)*	0.1 (0.2)*
L	0.3 (0.6)	0.3 (0.7)	0.1 (0.2)*	0.1 (0.2)*

Values are reported as mean (SD).

* *P* < 0.05 vs. 24 h.

### H-reflexes and M-responses

**Figure 2:**
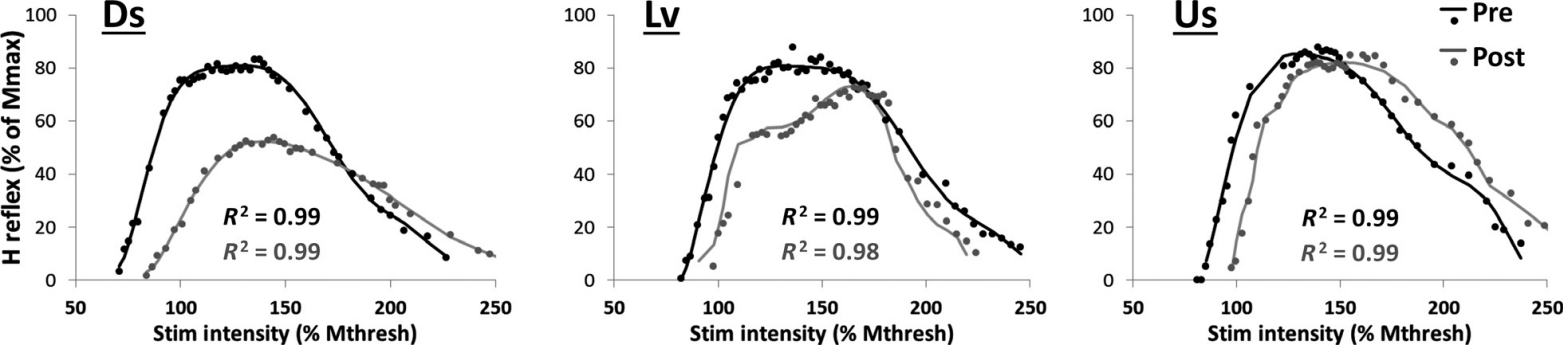
Representative H-reflex curves collected before (Pre, black trace) and after (Post, gray trace) 20 min of downslope (Ds), level (Lv), and upslope (Us) treadmill walking. Stimulus intensities are expressed as a percentage of the first stimulus intensity at which the M-response appeared (*x*-axis). H-reflexes are expressed as a percentage of the maximum M-response (*y*-axis). Solid lines represent the polynomial fit and dots represent raw data. R-squared values that represent the fit of the polynomial transformation with the raw data are shown. For this subject the peaks of the Pre curves were similar across days. The peak of the curves were virtually identical before and after Us walking. For this subject effects emerge for Ds and Lv walking, after which there was a reduction in the maximal amplitude of the curve.

Changes in H-reflex amplitude occurred as a result of Ds and Lv walking. In Fig.2 H-reflex curves from a representative subject are expressed as a percentage of the maximum M-response for pre and post Ds, Lv, and Us walking. The average *M*_max_ and *H*_max_ values across participants for Ds, Lv, and Us walking are shown in Table3. Pre *M*_max_ and *H*_max_ values did not change across days and were very reliable (*M*_max_, ICC = 0.97; *H*_max_, ICC = 0.94; *H*_max_/*M*_max_ = 0.95). For *H*_max_ the model interaction term was statistically significant (*P* = 0.02), and there was a statistically significant effect (Post-1 vs. Pre) for Ds and Lv walking only (*P* < 0.001 for Ds walking, and *P* = 0.02 for Lv walking). The change in *H*_max_ after Ds walking was larger than the change after Lv walking (36.5.2 ± 20.0% vs. 8.4 ± 17.0%, mean ± SEM, *P* = 0.03). The interaction term was not statistically significant for *M*_max_.

**Table 3:** Maximum H-reflexes and M-responses (peak-to-peak amplitude, mV)

	Ds	Lv	Us
*H*_max_			
Pre	4.2 (2.6)	4.7 (2.9)	4.6 (2.9)
Post-1	2.7 (2.0)*	4.4 (2.7)*	4.4 (3.0)
Post-2	3.3 (2.8)	4.3 (2.9)	3.8 (3.0)
*M*_max_			
Pre	7.2 (2.1)	7.3 (1.7)	7.3 (1.9)
Post-1	6.9 (2.4)	7.4 (1.9)	7.1 (2.1)
Post-2	6.4 (2.7)	7.2 (2.1)	7.0 (2.1)

Values are reported as mean (SD).

* *P* < 0.05 vs. Pre.

**Figure 3:**
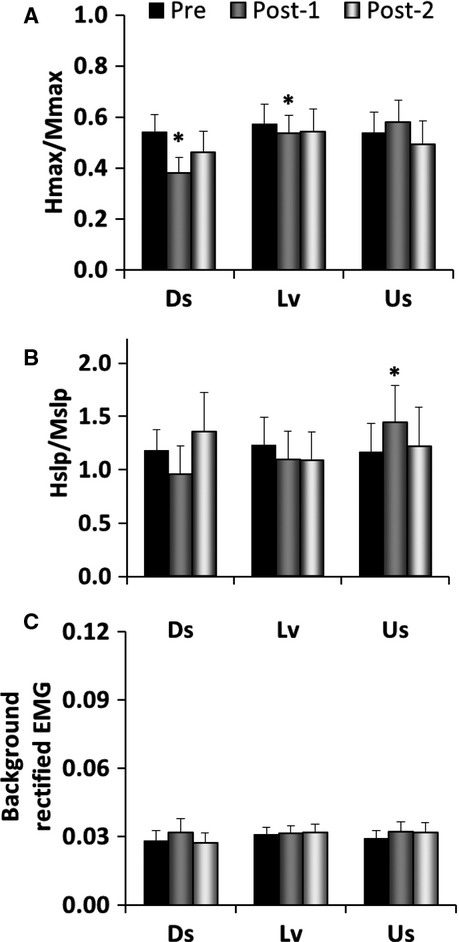
H-reflex results before and after 20 min of downslope (Ds), level (Lv), and upslope (Us) treadmill walking. (A) *H*_max_/*M*_max_ ratio, mean + SE, *P < 0.05 versus pre. There was a significant reduction after Ds walking and after Lv walking. (B) Hslp/Mslp ratio, mean + SE, *P < 0.05versus pre. There was a significant increase after Us walking. Prestimulus background EMG activity (100 msec prior to electrical stimulation) (C). There was no change in prestimulus background EMG activity across time.

*H*_max_/*M*_max_ ratios are illustrated in Fig.3A. The model interaction term was statistically significant for *H*_max_/*M*_max_ (*P* = 0.01). There was a reduction in *H*_max_/*M*_max_ for Post-1 versus Pre for both Ds walking (*P* < 0.001), and Lv walking (*P* = 0.02). The change after Ds walking was larger than the change after Lv walking (29.3 ± 6.2% vs. 6.8 ± 5.2%, mean ± SEM, *P* = 0.02). EMG biofeedback was used to standardize background EMG activity during the collection of H-reflex recruitment curves. As a result, background EMG activity did not change across bouts of testing (Fig.3C). Therefore, changes in background motor neuron recruitment can be ruled out as a cause of changes in H-reflex size. Results for Hslp/Mslp ratios are illustrated in Fig.3B. The model interaction term was statistically significant for Hslp/Mslp (*P* = 0.05). There was an increase in Hslp/Mslp for Us walking only (Post-1 vs. Pre, *P* = 0.04). Therefore, when spinal excitability was characterized using the Hslp/Mslp method, Us walking had an effect that was not detected using the *H*_max_/*M*_max_ approach.

### Rate-dependent depression

**Figure 4:**
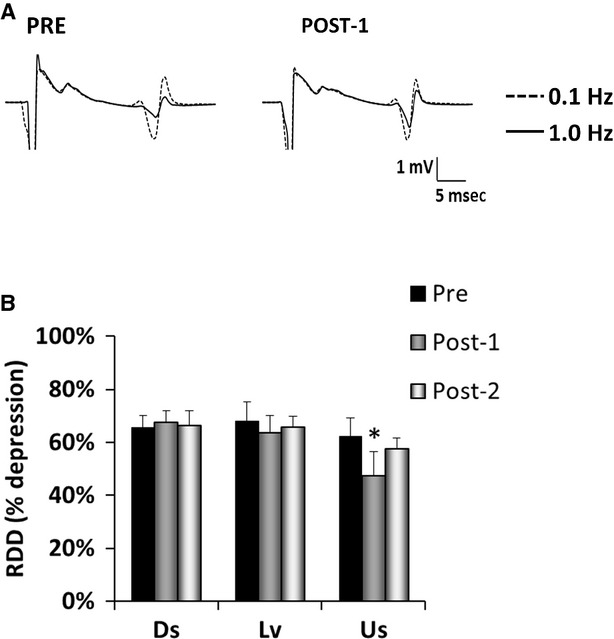
Rate-dependent depression (RDD) of the H-reflex before and after downslope (Ds), level (Lv), and upslope (Us) treadmill walking. (A) The average of 10 sweeps at 0.1 Hz (dotted line) and at 1.0 Hz (solid line), pre and post walking for one representative subject. Average H-reflex size was similar for 0.1 Hz, but depression with 1.0 Hz stimulation frequency was less at Post. (B) Average RDD results expressed as percent depression (i.e., % depression when H-reflexes were elicited at 1 Hz vs. when they were elicited at 0.1 Hz), mean + SE, *P < 0.05versus pre. There was a reduction in RDD after Us walking.

The final objective of this study was to evaluate the effect of slope walking on RDD (Fig.4). There was no detectable background EMG activity during the 100 ms preceding electrical stimulation (data not shown). This is consistent with the study protocol as subjects were asked to keep the leg at complete rest. RDD is expressed as a percentage (i.e., % depression when H-reflexes were elicited at 1 Hz vs. when they were elicited at 0.1 Hz) in Fig.4B. The model interaction term was statistically significant for RDD (*P* = 0.01). There was a significant reduction in RDD after Us walking (Pre vs. Post-1, *P* = 0.04), but there were no changes after Ds or Lv walking (*P* ≥ 0.14). Therefore, Us walking results in a transient reduction in the ability to diminish afferent input from Ia afferents with repeated activation.

## Discussion

This is the first study to evaluate the potential for slope walking to evoke spinal cord plasticity. Slope was used as a way to change the patterns of sensory, motor, and spinal inter-neuronal activity occurring during walking. The primary findings were that both Ds and Lv walking decreased spinal excitability, that this effect was significantly larger for Ds walking, and that this effect correlated well with physical activity level when elicited with Ds walking. Furthermore, Ds walking evoked a relatively minor cardiovascular response and perception of effort. This study also found that Us walking increased spinal excitability and caused a transient reduction in RDD. These observations expand our knowledge of the potential for spinal function to be modulated by a fundamental movement activity when the biomechanics have been altered to change the patterns of neural activity.

### Effects of downslope walking

The *H*_max_/*M*_max_ ratio in this study was depressed significantly more following Ds walking than following Lv walking. The *H*_max_/*M*_max_ ratio has been used in previous studies to evaluate short-term spinal plasticity. For example, acute loaded and unloaded cycling exercise causes reduced *H*_max_/*M*_max_ in healthy individuals (Motl and Dishman 2003; Motl et al. 2003a). It has also been reported that when cycle ergometry involves a motor skill component (e.g., variable resistance (Mazzocchio et al. 2006) or visuo-motor challenge (Perez et al. 2005)) there is significantly more reduction in *H*_max_/*M*_max_. Therefore, acute exercise can cause H-reflex depression, but the likelihood of such an effect is greater if the exercise involves more motor complexity. Results from this study show that a similar pattern occurs with walking. This might suggest that Ds walking involves more motor complexity as it was found here to result in more H-reflex depression. Furthermore, this study is not the first to find an effect of walking on H-reflexes. A recent study found that 30 min of Lv treadmill walking caused H-reflex depression (Thompson et al. 2006). Our study adds that even as little as 20 min of Lv treadmill walking depresses H-reflexes.

Two previous studies evaluated the effect of running on H-reflexes (Bulbulian and Bowles 1992; Racinais et al. 2008). Both found H-reflex depression that was more pronounced with a higher running intensity. Upslope walking (which was more intense than either Lv or Ds walking) did not cause a decrease in the *H*_max_/*M*_max_ ratio in this study. This suggests that the propensity for higher intensity locomotion to reduce *H*_max_/*M*_max_ is limited to running. However, Bulbulian and colleagues (Bulbulian and Bowles 1992) also reported a larger reduction in *H*_max_/*M*_max_ for 20 min of Ds (−10%) than for Lv running, both at 50% VO_2_max. The results of this study make it is clear that the effect of using a negative slope for running also applies to walking, despite numerous differences in neural control and mechanics between these two forms of locomotion (Cappellini et al. 2006).

This study also found that participants who reported more PA had a larger reduction in the *H*_max_/*M*_max_ ratio after Ds walking. This supports the idea that there are chronic central nervous system adaptations related to increased PA levels (Adkins et al. 2006), and also that such adaptations predispose healthy adults to the Ds walking effect discovered in this study. Highly trained cohorts have been found to present with smaller or larger resting H-reflexes than untrained cohorts, depending on the nature of their training. For instance, competitive power athletes (Casabona et al. 1990; Maffiuletti et al. 2001) and ballet dancers (Nielsen et al. 1993) have smaller H-reflexes, and endurance athletes have larger H-reflexes (Casabona et al. 1990; Maffiuletti et al. 2001). These differences have been attributed to the level of motor skill involved in these athletes’ competitive physical activities. However, highly trained cohorts as these were not evaluated in this study. Indeed, in this study there were no correlations between PA and any baseline measures of the H-reflex pathway. As this study is the first to report a decrease in spinal excitability with Ds walking, it is not clear if these very unique athletic populations would respond in unique ways to the Ds walking stimulus. We would hypothesize that there may be subtle differences in the amounts of skilled activity our participants regularly undertake that may have been captured in self-reports of more PA. However, it is not possible to quantify such potential differences with the PA questionnaire used in this study. Future studies could evaluate the effect of slope walking in distinct athletic populations to determine if high volumes of PA involving more or less motor control impact the slope-walking response. Such investigations could provide insight into the physiological basis of the slope-walking effects.

### Effects of upslope walking

This study found that Us walking increased the Hslp/Mslp ratio and reduced RDD. Increased effort may have prompted these effects as systolic blood pressure, heart rate, and RPE were elevated during Us walking. Increased MU recruitment and effort associated with Us walking may have resulted in increased serotonergic signaling from neurons of the raphe neuclei of the brain stem (Mazzardo-Martins et al. 2010) which have excitatory projections on alpha motoneurons (Barasi and Roberts 1974; White and Neuman 1980). Activity in the descending serotonergic system has been shown to increase in proportion to motor output (Jacobs et al. 2002). Furthermore, Cardona and Rudomin (Cardona and Rudomin 1983) reported a decrease in RDD in response to activation of brainstem serotonergic pathways in the isolated frog preparation. Norepinephrine system activity may have a similar effect as it also projects monosynaptically to motoneurons throughout the spinal cord (Holstege and Kuypers 1987) and increases with increased arousal (Aston-Jones et al. 2000). Both serotonin and norepinephrine enhance the effects of excitatory inputs to spinal motoneurons, producing long-lasting changes in motoneuron excitability (White and Neuman 1980). Therefore, in this study the motoneuron pool may have been less dependent on Ia synaptic transmission to reach firing threshold as a result of Us walking. This would facilitate a more sustained response to repetitive afferent inputs.

Decreased RDD resulting from Us walking as found in this study suggests the overall pattern of MU recruitment and incoming sensory information during Us walking results in a change in gaiting of afferent feedback that delegates more control of movement to peripheral reflex pathways. The increase in Hslp/Mslp found after Us walking would also contribute to this neural strategy. This is consistent with the idea that Us walking in healthy adults involves little skill development or motor learning. Rather, Us walking is unique in that it is associated with more MU recruitment (Gregor et al. 2006; Franz and Kram 2012) and force related feedback compared to Lv or Ds walking (Gregor et al. 2006). As such, a decrease in RDD could support an overall strategy to optimize muscle stiffness during Us walking and improve force transmission across skeletal muscle.

In conclusion, this study provides the first evidence that in healthy human participants walking for 20 min at a slow speed, treadmill slope determines the nature of resulting changes in the H-reflex pathway. Although speculative at this time, it is possible that these effects constitute the initial stage of activity-dependent spinal plasticity. Future studies should determine if repeated exposures of either Ds or Us walking convert the outcomes reported here into more permanent adaptations, as happens with motor or exercise training.
